# Renal Crisis as the Initial Manifestation of Scleroderma

**DOI:** 10.7759/cureus.25856

**Published:** 2022-06-11

**Authors:** Ronak Gandhi, Aparna Das, Daniel Gonzalez, Vijaya Murthy

**Affiliations:** 1 Internal Medicine, University of Texas Medical Branch at Galveston, Galveston, USA; 2 Rheumatology, University of Texas Medical Branch at Galveston, Galveston, USA

**Keywords:** autoimmune, connective tissue disorder, blurry vision, hypertensive urgency, rheumatology, systemic scleroderma, scleroderma renal crisis

## Abstract

We report the case of a young Hispanic woman who was originally admitted to the emergency department following hypertensive urgency and right-sided blurry vision. The patient did not carry a diagnosis of scleroderma at the time of the visit. However, upon further evaluation, the patient was found to have a scleroderma renal crisis. An angiotensin-converting enzyme (ACE) inhibitor was initiated promptly with subsequent normalization of the blood pressure and creatinine level. Scleroderma renal crisis is a rare, highly feared complication of scleroderma that if left untreated can be life-threatening. Therefore, it is important to identify this condition early and initiate therapy without delay.

## Introduction

Scleroderma is a chronic autoimmune disorder that carries a wide clinical presentation and is associated with multisystem involvement depending on the severity of the disease process [1,2]. Generally, both symptoms and hemodynamic instability help classify the disorder into the appropriate subtype, ranging from a relatively benign condition such as localized scleroderma to the more severe form of diffuse cutaneous systemic sclerosis [2]. Laboratory data, including platelet levels, inflammatory markers, blood urea, and creatinine levels, as well as serologies, such as antinuclear, anticentromere, anti-topoisomerase, and anti-RNA-polymerase III antibodies, are useful as they help characterize the severity of the condition. Diffuse cutaneous systemic sclerosis is the subtype most susceptible to complications, leading to multiorgan dysfunction [1-4]. Although gastrointestinal, pulmonary, and cardiac diseases can develop, the most concerning complication is scleroderma renal crisis. This is associated with sudden-onset renal disease and can quickly progress to renal failure if left untreated [1,4].

Despite scleroderma renal crisis typically taking years to present following the onset of systemic sclerosis, we present a case of a patient who developed scleroderma renal crisis despite no prior diagnosis of systemic sclerosis.

## Case presentation

A 36-year-old Hispanic woman was admitted following elevated blood pressure and right-sided blurred vision. She reported having light-colored patches on her back, chest, and shin that she first identified a year ago. She started noticing puffiness of her fingers and thickening of the skin of her hands two months before her presentation. Her other symptoms included arthralgia of the hands, dyspnea on exertion, Raynaud’s phenomenon, and dysphagia. 

Examination revealed blood pressure of 221/139 mmHg and tachycardia with a heart rate of 107 beats per minute. She was saturating well on room air and was afebrile. Further inspection revealed puffiness of her bilateral metacarpophalangeal joints, as well as digital pitting ulcers on her right thumb and left index finger. She also had thickening of the skin of her face and hands up to the level of her forearm, a hypopigmented macular rash on her right shin, telangiectasias on her face, and a “salt and pepper” appearance on her back.

Laboratory data revealed hemoglobin of 8.9, platelet count of 107,000, and creatinine of 1.38. Ophthalmology evaluation found multiple cotton-wool spots, single disc hemorrhage, and mottling along superior arcade on digital fundus exam, with visual acuity of 20/30 in the right eye. Serologies were positive for anti-nuclear antibody 1:1280 and nuclear pattern, with positive anti-topoisomerase (Scl 70), and negative RNA polymerase III antibody. The patient was subsequently diagnosed with systemic sclerosis complicated by scleroderma renal crisis. The patient underwent a chest X-ray which revealed minimal pericardial effusion, and a transthoracic echocardiogram (TTE) which showed no evidence of tamponade or pulmonary artery hypertension. Amidst her renal crisis, a barium swallow showed findings suggestive of an underlying motility disorder. The patient was started on captopril 25 mg three times per day. There was an initial elevation of creatinine which later normalized. The patient’s blood pressure was controlled after the initiation of captopril. Her right eye vision improved as well, with clearance of the single-disc hemorrhage on the digital fundus exam, and visual acuity of 20/20 in the right eye. She was discharged from the hospital with follow-up arrangements in a rheumatology clinic and outpatient pulmonary function testing.

**Figure 1:**
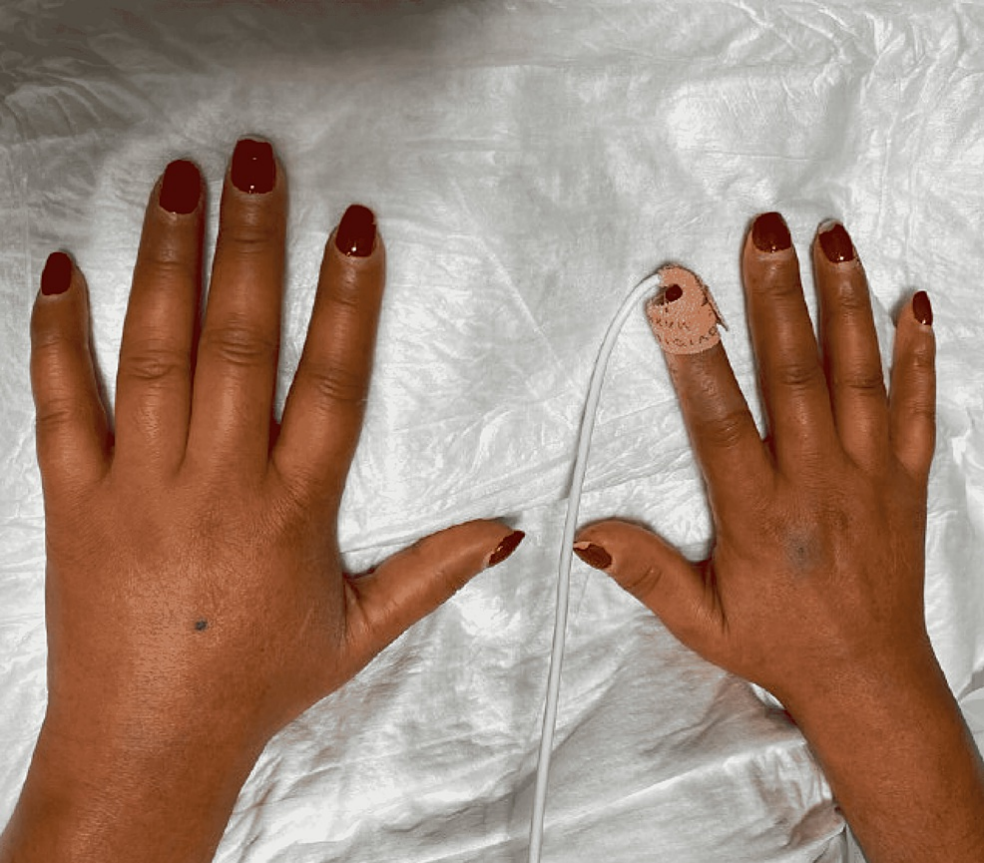
Puffiness of metacarpophalangeal joints and tapering of fingers

**Figure 2:**
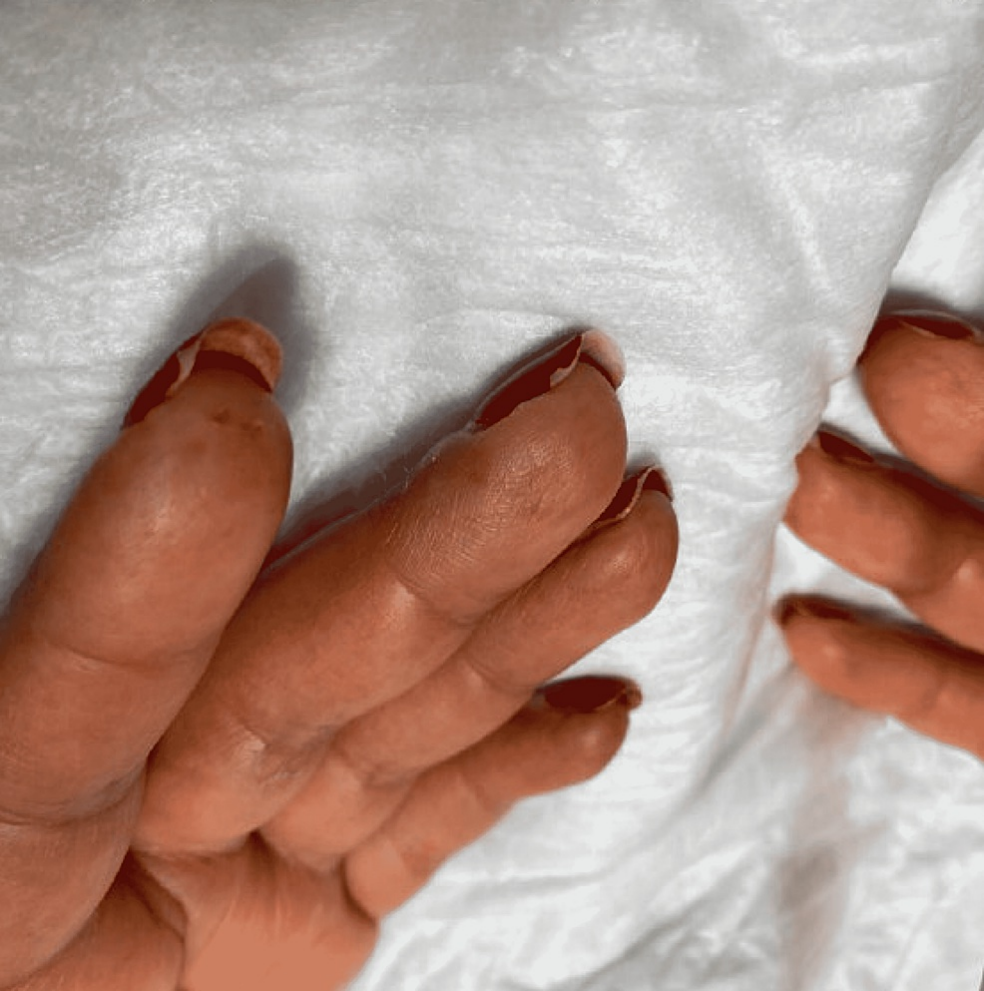
Digital pitting ulceration on left index finger

**Figure 3:**
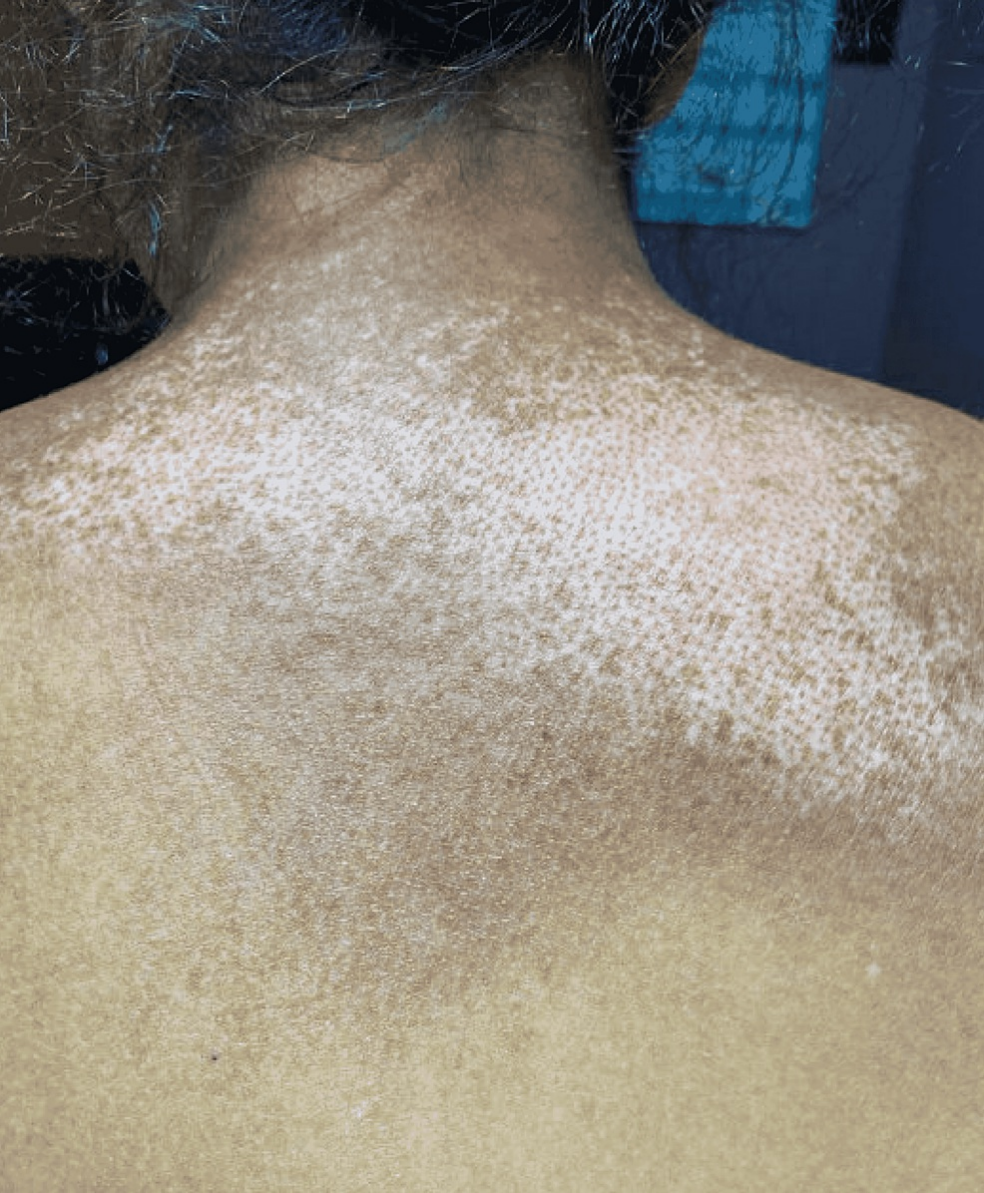
Hypopigmentation rash in a "salt and pepper" appearance on patient's back

## Discussion

The patient in this clinical vignette was identified as having scleroderma renal crisis despite no previous diagnosis of systemic sclerosis. Given her significantly elevated blood pressure with associated visual deficits, as well as elevated creatinine and new-onset proteinuria, the patient warranted hospitalization for additional workup. She was a relatively healthy young female with no autoimmune history. Despite carrying a history of hypercoagulability in the setting of multiple miscarriages, protein S deficiency, and methylenetetrahydrofolate reductase (MTHFR) gene mutation, there was low clinical suspicion for antiphospholipid syndrome. Further examination throughout the patient’s admission revealed progressively worsening severe skin changes, thrombocytopenia, and hypertensive retinopathy. Positive serologies, combined with physical exam findings drew concern for systemic sclerosis and subsequent scleroderma renal crisis in the setting of hypertension and renal involvement.

Scleroderma is a rare autoimmune connective tissue disorder that causes widespread collagen deposition, leading to progressive fibrosis of the skin, viscera, and vasculature [1,2]. Despite inadequate information regarding the full extent of its etiology, it is believed that both vascular processes and immunological events are critical to the development of scleroderma [5,6]. Immune mediators, such as cytokines, activate fibroblasts through their interactions with vascular endothelial cells and lymphocytes leading to excessive extracellular matrix formation [6,7]. In addition, recent evidence has emphasized the importance of reactive oxygen species and cellular stress in the production of the extracellular matrix via its effects on fibroblasts [5,6]. The epithelial-mesenchymal transition, which is vital in the development of scar tissue in both the lungs and kidney, is directly related to the extent of hypoxia and oxidative stress [6].

Due to the extensive pathology and various etiologies of systemic sclerosis, patients can present with a wide range of clinical features. For this reason, many subsets have been developed to help differentiate the disease process based on key features as well as the extent of organ involvement [2,3]. Localized scleroderma, otherwise known as morphea, is often considered the most benign of the systemic sclerosis subsets. It is a distinct disease that is limited primarily to the skin and underlying tissues without systemic organ involvement [2,8]. Patients with limited cutaneous systemic sclerosis (lcSSc) begin to develop significant vascular manifestations, in addition to their existing cutaneous findings. Such vascular features include telangiectasia and Raynaud’s phenomenon [2,3]. Cutaneous findings identify sclerosis of the skin localized specifically to the distal extremities [3,8]. LcSSc is often called CREST syndrome due to the common constellation of symptoms that it presents with - calcinosis cutis, Raynaud’s phenomenon, esophageal dysmotility, sclerodactyly, and telangiectasia [2,3,8].

Diffuse cutaneous systemic sclerosis (dcSSc), on the other hand, is considered the more severe form, as it is associated with more severe complications and a faster disease progression [1,4]. Cutaneous findings here involve sclerosis of the proximal extremities and trunk, and the disease process is often seen with multiorgan involvement, including fibrosis and hypertrophy of the lung walls, myocardium, and pericardium [1,9]. Pulmonary arterial hypertension (PAH) is a complication identified in both lcSSc and dcSSc and carries a high mortality rate [4,9].

Since not all patients fall within these classifications, two additional, less common subsets exist. Sine scleroderma identifies the group of patients who present with many of the clinical features of lcSSc or dcSSc without the cutaneous findings. The patients who present with signs of systemic sclerosis but also have features that identify with another rheumatological condition are diagnosed with systemic sclerosis overlap syndrome. To help further distinguish patients with concerns for systemic sclerosis objectively, the American College of Rheumatology (ACR) paired up with the European League Against Rheumatism (EULAR) in 2013 to create new classification criteria for diagnosing systemic sclerosis [1,3,4,10].

In patients with systemic sclerosis, especially dcSSc, complications can occur and many other organs can become involved [1-4]. Renal manifestations have been commonly associated with systemic sclerosis, affecting nearly 50% of patients. Evidence has shown that about 20% of cases progress to scleroderma renal crisis (SRC), most predominantly in patients who show signs of early diffuse scleroderma, rapidly progressing thickening of the skin, and palpable tendon friction rubs [11-14]. However, other factors including sepsis, dehydration, use of corticosteroids or cyclosporine, and positive serologies in anti-RNA polymerase III and anti-centromere have also been shown to contribute to patients’ increased risk of SRC [14,15].

Clinically, patients develop acute kidney failure with proteinuria and hypertensive urgency. Due to the severity of the patient’s hypertension, complications from this can arise, including ophthalmologic changes leading to blurry vision and hypertensive retinopathy, as well as neurologic involvement causing generalized seizures and hypertensive encephalopathy. With its vascular and autoimmune nature, SRC has been found to cause microangiopathic hemolytic anemia and thrombocytopenia [12,13]. Pathologically, vasculature within the glomeruli is affected, leading to intimal hyperplasia and a concentric “onion-skin” hypertrophy which can be seen on kidney biopsy. However, these findings are nonspecific and can be found in many other renal disorders, making a biopsy less useful [16].

Given scleroderma’s varying presentation, treatment revolves around symptom- and organ-specific management [1,2]. Systemic immunosuppressive therapy may be required in patients who have a more severe cutaneous disease or multiorgan complications in an attempt to slow down the disease progression. Those patients with severe skin fibrosis but minimal to no visceral organ involvement would benefit from methotrexate or mycophenolate mofetil [2,8,17]. Regarding those patients who have developed SRC, the main goal is to control their hypertension. ACE inhibitors serve as the main antihypertensive treatment given their added value in preserving kidney function [15]. Specifically, captopril has been identified as the preferred ACE inhibitor as it carries a rapid onset of action and is short-acting, allowing for more flexibility in dose adjustments to control the patient’s blood pressure more appropriately. Even in those with normotensive SRC, captopril is efficacious at low doses given its renal advantages. Despite its similarities with angiotensin receptor blockers (ARBs), ARBs have not been found to show similar benefits in SRC. ACE inhibitors should be given immediately once hypertension has been identified in the setting of systemic sclerosis; however, it is important to note that pre-emptive treatment or prophylaxis with ACE inhibitors in such patients before changes in blood pressure or kidney function has been associated with increased mortality and should be avoided. In patients with resistant hypertension, it is recommended to add a calcium channel blocker, which should also assist in managing the patient’s Raynaud’s phenomenon. However, beta blockers are contraindicated given their risk of vasospasm and should not be given [15,17,18].

Despite the benefits of ACE inhibitors in SRC, the condition still carries a poor prognosis. A systematic review of current literature has shown that in individuals with systemic sclerosis that progresses to SRC (not including end-stage renal disease), the estimated survival rate at the time of SRC onset is about 80% at six months, 70% at one year, and 60% at three years [19]. Patients with SRC who fail treatment or are left untreated have a high likelihood of progression to end-stage renal disease [4,17,18]. About 30% of patients who fail ACE inhibitor treatment end up requiring dialysis, whereas roughly 10% of patients undergo renal transplants [19]. However, survival rates are significantly better (73% compared to 44%) in patients who have undergone renal transplants compared to those on dialysis. Thus, current recommendations advise pursuing a renal transplant if a patient’s kidneys fail to recover after one to two years of dialysis [19].

## Conclusions

Although renal complications can occur following the onset of dcSSc, it commonly takes a few years before a patient’s renal injury advances to SRC. However, as evidenced by this case, it is important to keep SRC on the differential even if the patient is without a known history of systemic sclerosis. Patients with severe hypertension, especially those with associated multisystem complications, can require extensive workup to determine the underlying etiology. Given its life-threatening nature, clinicians should have a low threshold for diagnosing SRC in the setting of renal manifestations and elevated blood pressure, and a rapid yet decisive treatment plan should be initiated.
